# Novel Anti-arrhythmic Medications in the Treatment of Atrial Fibrillation

**DOI:** 10.2174/157340312803760785

**Published:** 2012-11

**Authors:** Pradyot Saklani, Allan Skanes

**Affiliations:** The University of Western Ontario, Arrhythmia Service, Division of Cardiology, London, Ontario, Canada

**Keywords:** Atrial fibrillation, anti-arrhythmic drugs, atrial selectivity, dronedarone, novel agents, ranolazine, vernakalant.

## Abstract

Atrial fibrillation (AF) is a prevalent condition particularly amongst the elderly, which contributes to both morbidity and mortality. The burden of disease has lead to significant increases in health care utilization and cost in recent years. Treatment of Atrial fibrillation consists of either a rate or rhythm control strategy. Rhythm control is achieved using medical management and/or catheter ablation. In spite of major strides in catheter ablation, this procedure remains a second line treatment of AF. Anti-arrhythmic medications represent the main treatment modality for the maintenance of sinus rhythm. Amiodarone has been used for decades because of its efficacy and lack of pro-arrhythmia despite numerous extra-cardiac side effects. Novel agents such as Dronedarone were designed to emulate Amiodarone without the extra-cardiac side effects. Unfortunately recent trials have raised concerns for the safety of this medication in certain patients. Other agents such as Vernakalant and Ranolazine are in development that promise to be more atrial selective in their action, thereby potentially avoiding pro-arrhythmia and heart failure side effects. It remains to be seen however if one or more of these agents achieves the required high efficacy and safety threshold. This review summarizes the main anti-arrhythmic clinical trials, early phase trials involving novel agents and examines the conflicting data relating to Dronedarone.

## NOVEL ANTI-ARRHYTHMIC MEDICATION IN THE TREATMENT OF AF

Atrial fibrillation (AF) is the most common arrhythmia requiring treatment. AF is not benign and is associated with significant morbidity and mortality [1, 2]. The prevalence of this condition increases with age and affects 9% of the population over the age of 80 [3]. The rates for hospitalization have trebled over the last 2 decades resulting in a substantial burden on the health care system [1].

The treatment of AF has evolved over the same period. The Atrial fibrillation Follow-Up Investigation of Rhythm Management (AFFIRM) and Rate Control versus Electrical Cardioversion for Persistent Atrial fibrillation (RACE) trials altered the treatment paradigm in Atrial fibrillation. Rhythm control was shown to offer no survival advantage or reduction in stroke rates over rate control in Atrial fibrillation [4, 5]. Furthermore there was a trend towards increased mortality, with greater rates of hospitalization and adverse drug reactions in the rhythm control group [5]. The Atrial fibrillation and Congestive Heart Failure (AF-CHF) trial demonstrated a similar lack of survival benefit or reduction in the rate of stroke in patients with congestive cardiac failure, poor left ventricular systolic function and intermittent AF randomized to a rhythm over rate control strategy [6]. At the same time, there have been tremendous advances in the development of catheter ablation techniques and technology. Catheter ablation for AF has demonstrated superior outcomes in maintenance of sinus rhythm, morbidity, cardiac function, exercise capacity and quality of life compared to treatment with Anti-arrhythmic therapy [7-10]. 

As a consequence of these advances, the impetuous for the development of newer anti-arrhythmic medications has been hampered. The following review summarizes the newest drugs that have been approved for the treatment of Atrial fibrillation, against the backdrop of established anti-arrhythmic drugs. (See Table **1**). Despite being much maligned due to an adverse side effect profile largely observed only with prolonged use at high doses, Amiodarone remains one of the most efficacious anti-arrhythmic medications used in the management of Atrial fibrillation. This was demonstrated in the Canadian Trial of Atrial fibrillation (CTAF), a landmark study that randomized patients with at least one episode of Atrial fibrillation in an unblinded fashion to Amiodarone, Sotalol or Propafenone [11]. The primary end point was the length of time to first recurrence of ECG-confirmed Atrial fibrillation. After a mean follow up duration of 16 months, the recurrence rate for Atrial fibrillation was almost double in the patients treated with Sotalol or Propafenone compared to Amiodarone (63% versus 35%, P<0.001). Sinus rhythm was maintained for one year in 39% and 69% (P<0.001) of patients assigned to Sotalol or Propafenone and Amiodarone respectively. The median time to first recurrence of Atrial fibrillation was 98 days in patients assigned to Sotalol or Propafenone and >468days in those assigned to Amiodarone. This effect of Amiodarone was consistent across a broad range of pre-specified subgroups including age, type of Atrial fibrillation and the presence of cardiovascular or structural heart disease. These striking results were observed with no significant difference in the rates of death or non-fatal major clinical events. Furthermore there were significantly lower rates of study drug discontinuation in the Amiodarone group (34% versus 46%, P<0.001). There was however a non-significant trend towards greater non-fatal adverse events in the Amiodarone group. While Amiodarone use appears safe in the short to intermediate term, reservations exist pertaining to major clinical side effects with long-term use. Nonetheless Amiodarone, by way of its high efficacy, remains the benchmark against which other drugs are compared. The goal for drug manufactures is to replicate the effectiveness and lack of pro-arrhythmia of amiodarone, whilst reducing the long-term side effect profile of any new agent.

## DOFETILIDE

Dofetilide is a novel anti-arrhythmic medication that has a pure Class III effect. Dofetilide selectively inhibits the rapid component of the delayed rectifier potassium current (I_Kr_) in a time and voltage dependent manner [12]. This results in prolongation of the action potential duration and the effective refractory period of cardiac myocytes, without affecting conduction velocities or contractility [13]. Like Amiodarone, it is the only other anti-arrhythmic medication that can be used safely in patients with impaired left ventricular systolic function and ischemic heart disease. A number of studies have demonstrated its efficacy in this setting [14-16]. The Danish Investigations of Arrhythmia and Mortality on Dofetilide in Congestive Heart Failure (DIAMOND-CHF) trial randomized patients with symptomatic congestive heart failure (CHF) and severe left ventricular systolic dysfunction (EF <35%) to oral Dofetilide or placebo [14]. After a median follow up duration of 18months, there was no difference in all cause mortality. However, treatment with Dofetilide reduced the risk of hospitalization (RR 0.75, P<0.001) with worsening heart failure irrespective of whether AF was present at baseline. A pooled substudy analysis of patients in the DIAMOND-CHF and DIAMOND-MI studies with Atrial fibrillation/flutter, poor left ventricular systolic function (EF<35%) and either recent CHF or MI, showed that Dofetilide maintained sinus rhythm at 1 year in 79% of patients compared to 42% in the placebo treated group (P<0.001) [16]. In spite of the lower than expected AF/flutter recurrence rates in the placebo treated group, the rate of AF/flutter recurrence was approximately one third in the patients randomized to Dofetilide. Rates of all cause (RR 0.7 P<0.005) and heart failure related hospitalizations (RR 0.69 P<0.02) were lower in the Dofetilide compared to placebo treated patients. The Symptomatic AF Investigative Research on Dofetilide (SAFIRE-D) trial found that 58% of patients with AF/flutter treated with Dofetilide were maintained in sinus rhythm at 1 year compared to 25% of patients treated with placebo (P=0.001) [17]. The median time to recurrence was increased from 27 days to >365 days in patients treated with Dofetilide compared to placebo [17]. 

Dofetilide is well tolerated, with few side effects. The main clinically severe adverse effect is QT prolongation and torsade de pointes (TdP), a life-threatening ventricular arrhythmia. TdP was observed in 1.2-3.3% of patients treated with Dofetilide [14-17]. The majority of such cases occurred within the first 72 hours of initiation of Dofetilide. As a precaution therefore Dofetilide should always be initiated in hospital with 72 hours of continuous cardiac monitoring and daily measurement of the QT interval. Dofetilide is renally excreted and requires appropriate dose adjustment in patients with reduced GFR. Female sex and NYHA class III or IV were associated with a significantly greater risk of TdP (OR 3.2 and 3.9 respectively) even after dose adjustment for creatinine clearance [14]. The clinical utility of Dofetilide has been greatly limited by concern of TdP and the requirement of hospitalization for its initiation.

## DRONEDARONE

Dronedarone is one of the newest anti-arrhythmic approved for the treatment of AF since Dofetilide was introduced 13 years ago. Dronedarone is a multi-channel blocker with an electrophysiological profile similar to Amiodarone. It has anti-adrenergic properties, while also inhibiting multiple trans-membrane ion channels including potassium (I_Kr_, I_Ks_, I_K1_, I_to_), sodium (I_Na_) and L-type calcium channels (I_Ca-L_) [18].

Dronedarone is structurally modeled on Amiodarone. It was altered biochemically in an attempt to retain the potent anti-arrhythmic effect of Amiodarone without the toxicity. Both agents are benzofuran derivatives. Dronedarone lacks the iodine moiety that is responsible for thyroid and other possible organ toxicity associated with Amiodarone use [18]. In the case of Dronedarone, there has been no significant difference in the incidence of pulmonary toxicity compared to placebo in any of the trials using this agent [19-22]. The true incidence of pulmonary toxicity with Dronedarone however, is difficult to estimate since these side effects are rare and ordinarily only seen after several years of Amiodarone use. Dronedarone is also less lipophilic than Amiodarone as a result of the addition of a methyl-sulfonamide group. As a consequence, neurotoxicity is diminished. The decreased lipophilicity also contributes to its short half-life of approximately 24 hours by shrinking Dronedarone’s volume of distribution. Dronedarone is metabolized by the hepatic enzyme cytochrome P-450, 3A4 isoform. It raises digoxin levels when used concomitantly but does not interfere with warfarin metabolism. Dronedarone, like amiodarone, reversibly inhibits the tubular excretion of creatinine, raising serum levels by approximately 18% without interfering with the glomerular filtration rate [23]. Diarrhea and nausea are common side effects of Dronedarone encountered on average in approximately 8% and 5% of treated patients respectively [19-21].

### Positive trials of Dronedarone in Atrial Fibrillation

EURopean trial In Atrial fibrillation or flutter patients receiving Dronedarone for the maintenance of Sinus rhythm (EURIDIS) and American-Australian-African trial with Dronedarone In Atrial fibrillation or flutter patients for the maintenance of Sinus rhythm (ADONIS) were the initial phase III trials conducted, testing the efficacy of Dronedarone in maintaining sinus rhythm [24]. These identical double blind randomized placebo controlled trials were conducted in Europe and selected non-European countries (dominated by North America) respectively. The primary endpoint of these trials was time to first recurrence of Atrial fibrillation. Pooling the data together from the two trials demonstrated that time to first recurrence of Atrial fibrillation was significantly longer in the Dronedarone arm at 116 days compared to 53 days in the placebo arm. At 12 months, the rates of recurrence of Atrial fibrillation were 64.1% and 75.2% (HR 0.75, P<0.001) in the Dronedarone and placebo arms respectively. When Atrial fibrillation did recur, the ventricular rate in the Dronedarone group was lower than the placebo group by 13.7bpm (P<0.001). Post hoc analysis also revealed a reduction in combined mortality and hospitalization with a Hazard ratio of 0.73 (P=0.01) in favor of Dronedarone. These benefits of Dronedarone were observed in the absence of any significant ventricular arrhythmia, thyroid, hepatic, pulmonary or other organ toxicity, albeit over a relatively brief follow up period of 1 year. 

Based on the post-hoc analysis of EURIDIS/ADONIS, the ATHENA (A placebo-controlled, double-blind, parallel arm Trial to assess the efficacy of Dronedarone 400 mg bid for the prevention of cardiovascular Hospitalization or death from any cause in patiENts with Atrial fibrillation/Atrial Flutter) study was designed to prospectively test the effects of Dronedarone on mortality and hospitalization. The population included patients at risk of cardiovascular events due to at least one of the following: age greater than 70 years, hypertension, diabetes mellitus, prior TIA, stroke or systemic thromboembolic event, EF <40%, or left atrial diameter ≥50mm [19]. This multicenter, double blind randomized controlled trial observed a 24% relative risk reduction (P<0.001) for the combined primary outcome of all cause mortality and first hospitalization due to cardiovascular events. The favorable primary outcome was driven largely by a reduction in rates for hospitalization due to AF in the Dronedarone group. There was no significant difference in the rates of hospitalization for heart failure, ventricular arrhythmia or non-fatal cardiac arrests. There was also an observed lower incidence of acute coronary syndrome in the Dronedarone group (HR 0.7, P=0.03). Cardiovascular mortality, specifically from malignant arrhythmia was significantly lower in the Dronedarone group (HR 0.71, P=0.03). Post hoc analysis demonstrated that Dronedarone reduced the risk of stroke by 34% (P=0.027) [25]. Whist historically considered intuitive, this was the first signal that an anti-arrhythmic used in the treatment of Atrial fibrillation was capable of reducing the risk of stroke. The mechanism of such a reduction in stroke risk however is likely to be more complex than simply maintenance of sinus rhythm alone [5]. Other proposed mechanism include the favorable modest but clinically significant blood pressure lowering effect of Dronedarone as well as avoidance of tachycardia induced hypotension due to the negative dromotropic effects of Dronedarone during paroxysms of Atrial fibrillation [25]. 

In ATHENA, the safety profile of Dronedarone was once again highlighted, with no major side effects observed. There was however a higher rate of gastro-intestinal upset, rash, bradycardia, QT prolongation and elevated serum creatinine in the Dronedarone group. One case of torsade de pointes was reported in the Dronedarone group. In the early stages of Dronedarone use, two associated cases of acute liver failure requiring transplantation were reported. No significant derangement of liver function associated with Dronedarone use has been observed in any of the major trials since, including in ATHENA. One major limitation of the study was the high rate of drug discontinuation (30.2% in the Dronedarone arm over a median follow up duration of 22 months) albeit not related to adverse effects. While such a high rate of drug discontinuation could have underestimated the benefit of Dronedarone, it could have similarly overestimated it, when an intention to treat analysis was performed. This may be relevant in the context of the conflicting results seen in the negative trials.

### Dronedarone in Patients with LV Dysfunction

The Diamond-CHF trial published in 1999 showed that the pure class III anti-arrhythmic agent Dofitilide reduced the rates of hospitalization for worsening heart failure in patients with prior congestive cardiac failure and severe impairment of left ventricular systolic function (EF ≤35%) [14]. Against the backdrop of this trial, Dronedarone was studied in a similar fashion in the Antiarrhythmic trial with Dronedarone in Moderate to severe congestive heart failure Evaluating Morbidity DecreAse (ANDROMEDA) study [22]. This was a double blind, randomized placebo controlled trial in patients recently hospitalized with congestive cardiac failure and severe impairment of left ventricular systolic function (EF ≤35%). The primary outcome was a composite of all cause mortality and hospitalization for heart failure. The study was terminated prematurely 7 months after commencing due to excess mortality in the Dronedarone group. Worsening heart failure contributed to the majority of the excess events. There was no significant difference between the two groups in the rates of arrhythmic or sudden death. After a median follow up of 2 months, the mortality in the Dronedarone and placebo groups were 8.1% and 3.8% respectively, yielding a hazard ratio of 2.13 (P=0.03). Post hoc subgroup analysis suggested that in the Dronedarone group, increasing severity of left ventricular systolic dysfunction was associated with greater mortality. Despite the limitations of post-hoc analysis, it was postulated that Dronedarone might have contributed either directly or indirectly to worsening heart failure in patients with depressed left ventricular systolic function. It should be pointed out that trials that are terminated prematurely have the potential to overestimate the treatment or harm effect. Such an argument however is harder to defend in the context of the adverse findings of the subsequent Permanent Atrial fibrillation outcome Study using Dronedarone on top of standard therapy (PALLAS) trial, which has once again cast doubt on the safety of Dronedarone particularly in patients with poor left ventricular systolic function.

### Dronedarone Versus Amiodarone

The Dionysos trial compared Dronedarone to Amiodarone in a randomized control trial of patients with persistent Atrial fibrillation with the primary composite endpoint of AF recurrence, or premature drug discontinuation because of intolerance (main safety endpoint) [20]. Dronedarone was significantly less effective at maintaining sinus rhythm following cardioversion compared to Amiodarone with recurrence rates of 36.5% and 24.3% respectively over median treatment duration of 7 months. There was no significant difference in the incidence of the main safety endpoints (the first occurrence of thyroid, pulmonary, hepatic, neurologic, skin, eye or GI specific events, or premature study drug discontinuation following an adverse event). However when GI events were excluded in a pre-specified analysis, concentrating on the more severe adverse events, a statistically significant (P=0.002) 39% relative risk reduction was observed in the main safety endpoint with Dronedarone.

### Dronedarone in Permanent Atrial Fibrillation

Based on the known pharmacological effects of Dronedarone, as well as its documented rate slowing from the EURIDIS/ADONIS study, Dronedarone was evaluated in patients with permanent Atrial fibrillation as a rate control agent. The Efficacy and safety of Dronedarone for The cOntrol of ventricular rate during Atrial fibrillation (ERATO) trial was a small placebo controlled RCT designed to assess the efficacy and safety of Dronedarone as a rate control agent in addition to standard therapy in patients with permanent Atrial fibrillation [26]. Compared to placebo, Dronedarone was shown to reduce the ventricular rate at day 14 by 11.7 beats per minute (P<0.001). The magnitude of effect was sustained at 6 months. The degree of rate control was even greater during exercise with a mean reduction of 24.5 beats per minute. This was achieved without compromising exercise tolerance.

The recently published PALLAS trial was a multicenter double blind randomized placebo-controlled trial to assess the effect of Dronedarone on cardiovascular morbidity and mortality in patients with permanent Atrial fibrillation [21]. The impetus for the study came from the favorable results of the ATHENA trial, where rates of death from cardiovascular causes, arrhythmic death, and stroke were significantly lower in those treated with Dronedarone, even in the subgroup (178 patients or 7.6% of the treatment arm) that developed permanent AF during the study [19, 25, 27]. It was postulated that the significant reductions in heart rate, blood pressure and arrhythmic death observed in patients treated with Dronedarone was responsible for the reduced events in those patients with permanent AF, where maintenance of sinus rhythm could play no role. Patients older than 65 years of age with permanent Atrial fibrillation and additional cardiovascular risk factors based on specific criteria were eligible for enrollment in PALLAS. The two co-primary endpoints were composites of 1) cardiovascular morbidity and mortality and 2) cardiovascular hospitalization and death respectively. The study was terminated prematurely for safety reasons. Unexpectedly high rates of heart failure episodes or hospitalization (HR 2.16, P<0.001), stroke (HR 2.32, P=0.02), and all cause mortality (HR 1.94, P=0.049) were observed in the group randomized to Dronedarone. As a consequence both co-primary outcomes were significantly higher in the Dronedarone group. The effects of Dronedarone were consistent across all subgroups even in those with NYHA class II symptoms and EF >40%. The conclusion drawn from the study, according to the authors, was that Dronedarone should not be used to treat patients with permanent Atrial fibrillation and cardiovascular risk factors.

### Analysis

How are we left to reconcile the seemingly disparate results of ATHENA and PALLAS? The mechanism for the different results is not obvious and sub-group analysis of the PALLAS study provides no insight. We are left to carefully analyze the differences in the study populations to try to understand in whom Dronedarone is safe to use and in whom it may be harmful. 

### Permanent Versus Intermittent Atrial Fibrillation

The most striking difference between the patients in the ATHENA and PALLAS trial was the pattern of Atrial fibrillation. Was the diametric response to Dronedarone in the two trials largely based on this interaction? This seems at odds with repeated trials that have shown that maintenance of sinus rhythm offers no mortality or morbidity advantage over a rate control strategy. Furthermore post hoc subgroup analysis of the ATHENA showed that patients who developed permanent Atrial fibrillation during the study had a similar benefit as those with intermittent Atrial fibrillation [19]. It seems likely then that the differences in co-morbidities played a more important role.

### Heart Failure

The use of anti-arrhythmic drugs, especially those with potent sodium channel blocking properties in the setting of poor left ventricular systolic function, with or without congestive cardiac failure has repeatedly been shown to be associated with increased mortality [22, 28, 29]. The increased mortality in the ANDROMEDA trial was predominantly due to worsening heart failure in the group treated with Dronedarone without an increase in arrhythmic death. In the ANDROMEDA and PALLAS trials there was an increase in the rates of heart failure events or hospitalizations. The exact mechanism by which Dronedarone may have contributed to these events is unknown. Certainly in the canine model, long term *in vivo* administration of Dronedarone did not produce a negative inotropic effect on post infarct myocardial function [30]. Similarly Dronedarone did not compromise left ventricular systolic function in patients with compensated heart failure and LVEF <30% [31].

Surprisingly, subgroup analysis of subjects in the PALLAS study demonstrated no interaction between LV dysfunction (greater or lesser than 40%) or NYHA class (II or III) and Dronedarone use with either of the co-primary outcomes or hospitalization for heart failure [21]. Regardless, there was a very strong signal for increased heart failure events in the PALLAS study, which was statistically highly significant. Subgroup analysis of subjects in the ANDROMEDA trial however found a significant interaction between poor LV function (wall motion index <1) and Dronedarone use with increased mortality. The reported p value of 0.04 should be interpreted with caution, as it is uncorrected for multiple tests of interaction [32].

The increased mortality in the ANDROMEDA trial was predominantly due to worsening heart failure without an increase in arrhythmic death. In contrast excess mortality in the PALLAS trial was attributed primarily to arrhythmic death. This may not be solely due to the use of Dronedarone in the presence of LV dysfunction and CHF. One hypothesis is the proposed metabolic interaction between Dronedarone and Digoxin.

### Digoxin Concentration

The increased rates of arrhythmic death in the PALLAS trial may be associated with elevated Digoxin levels through a P-glycoprotein interaction with Dronedarone [21]. Digoxin levels above 1.2ng/ml have been associated with greater cardiovascular mortality [33]. A third of patients in the PALLAS trial were reported to have an elevated Digoxin level within this range. This appears to be a plausible explanation for the difference in mortality between the ATHENA and PALLAS trials, where the prevalence of Digoxin use was double in the latter. In spite of this, the difference in the prevalence of Digoxin use amongst the trials is insufficient to explain the diametric response to Dronedarone. Furthermore subgroup analysis of Digoxin use in the PALLAS study found no interaction with either of the co-primary outcomes [21]. Additionally a similar prevalence of Digoxin use between the PALLAS and ANDROMEDA trials (~30%) was associated with contrasting rates of arrhythmic death. The smaller trial ERATO had an even higher prevalence of Digoxin use (43%) without any observed increased mortality in the treatment arm. Moreover Digoxin toxicity does not adequately explain the increased prevalence of stroke and heart failure seen in the PALLAS trial.

### Role for Dronedarone

On balance, the exact mechanism of increased adverse events in the PALLAS study remains unclear. Accordingly, a cautionary tone should be struck when discussing the use of Dronedarone in the treatment of AF. The Canadian Cardiovascular Society (CCS) has recently re-examined its 2010 Guidelines for the use of Dronedarone in patients with AF in a recent update [34]. They recommend that Dronedarone not be used in patients with permanent AF for the purpose of rate control. Likewise, Dronedarone should not be used in patients with a history of CHF or LV dysfunction and EF < 40%. Also Dronedarone should be used with caution in patients taking Digoxin. It is expected that both the European Society of Cardiology (ESC) and the American Heart Association / American College of Cardiology Guidelines will update their respective guidelines in the coming months, likely with similar restrictions for the safe use of Dronedarone. 

For whom is Dronedarone’s use reasonable? It seems reasonable to use Dronedarone in patients with paroxysmal or persistent AF and preserved LV function (with no history of CHF) to maintain sinus rhythm. Comparable to Amiodarone use, vigilance on the part of the clinician is warranted when prescribing Dronedarone. Clinicians should monitor their patients for early signs of heart failure. Similarly Dronedarone should be discontinued as soon as a rhythm control strategy is abandoned. It is advised that all patients taking Dronedarone should have liver enzymes assessed 6 months after initiation of the drug and preferably at baseline for comparison.

All Antiarrhythmic drugs have limitations, pertaining specifically to their propensity to cause side effects, pro-arrhythmia and increased mortality. Amiodarone is one of the least pro-arrhythmic drugs, but is nonetheless plagued by numerous extra-cardiac organ toxicities. Sotalol increases the risk of torsade de pointes, a life-threatening ventricular arrhythmia due to a long QT interval. Class Ic agents are contraindicated in patients with known coronary disease and also without preserved left ventricular systolic function due to their documented pro-arrhythmic effect. Not surprisingly therefore, Dronedarone also has limitations to its use in AF.

## VERNAKALANT

Most anti-arrhythmic medications alter the function of ion channels expressed in both atrial and ventricular tissue. Treatment of atrial arrhythmia thus gives rise to the potential for ventricular arrhythmia as a result of the inadvertent pro-arrhythmic changes to ventricular conduction velocities and refractoriness. Vernakalant is a novel anti-arrhythmic medication, which avoids such complications by being relatively atrial specific in its mechanism of action. Vernakalant blocks the function of I_Kur_, I_Kto_, I_KAch_ and I_Na. _[35] I_Kur_ is expressed only in atrial tissue and is an important ion channel responsible for the dominant repolarization current in the atria [35]. Vernakalant has been show *in vivo* studies to prolong atrial refractoriness, AV node conduction and refractoriness and QRS duration in a dose-dependent fashion [35]. It had no demonstrable effect on ventricular refractoriness.

Vernakalant is effective in converting recent onset (<7 days duration) and postoperative (CABG and/or valve surgery) Atrial fibrillation when administered intravenously as one or two brief 10min infusions as required. This result has repeatedly been demonstrated in placebo controlled randomized Phase II and III trials [36-38]. Approximately 50% of patients treated with IV Vernakalant reverted to sinus rhythm within a median duration of 8-14 minutes. This was a highly statistically significant result across the various trials compared to placebo. Vernakalant however was ineffective in converting AF of greater than 7 days duration or atrial flutter to sinus rhythm. Notably no clinically severe adverse effects or ventricular arrhythmia were observed in these trials. Building on the positive results seen in the placebo-controlled trials, the AVRO trial compared intravenous Vernakalant to Amiodarone in patients with recent onset (<48hours) Atrial fibrillation [39]. Amiodarone was chosen as a comparator because it is widely available and used in the acute conversion of Atrial fibrillation in many emergency departments. Rates of successful cardioversion (within 90 minutes) were approximately 10 times greater with Vernakalant than Amiodarone (51.7% Versus 5.2%, P<0.0001). 51.7% of patients treated with Vernakalant reverted to sinus rhythm within a median duration of 11minutes. No clinically significant adverse side effects, torsade de pointes or malignant ventricular arrhythmia were observed. Amongst patients who reverted to SR with Vernakalant, 98.3% were maintained at 4 hours. Vernakalant organized AF into Atrial flutter more frequently than Amiodarone (8.6% versus 0.9%) without any cases of 1:1 atrioventricular conduction during tachycardia.

An oral preparation of Vernakalant has been investigated most recently in a dose finding trial in patients with Atrial fibrillation scheduled for cardioversion [40]. Vernakalant was effective only at the highest dose of 500mg bid in maintaining SR post cardioversion. At this dose, Vernakalant delayed the median time to AF recurrence from 29 days (placebo) to >90 days (P=0.028). There was a 45% relative risk reduction (p=0.023) compared to placebo in the recurrence of symptomatic AF. 49% of patients treated with Vernakalant maintained sinus rhythm at 90 days compared to 36% of patients treated with placebo (P=0.021). In spite of a modest prolongation in the mean QTc (5.8ms), no pro-arrhythmia were observed in the Vernakalant group. The most frequent side effect encountered was bradycardia (2.7%).

Vernakalant is a promising new anti-arrhythmic medication, which appears to have an excellent safety profile. The intravenous preparation is effective in the cardioversion of acute Atrial fibrillation. More study is required into the efficacy of the oral preparation especially compared to other standard of care anti-arrhythmic medications used in AF. Longer duration of follow up and its impact on other clinical endpoints such as rates of stroke, heart failure and mortality need to be studied. Whereas IV Vernakalant has been approved for use in the acute cardioversion of Atrial fibrillation in Europe, the oral preparation remains an investigation drug.

## RANOLAZINE

Recently, an anti-anginal, Ranolazine, with selective atrial sodium channel blockade has been investigated for the conversion and prevention of Atrial fibrillation. Ranolazine appears to block a number of channels including late I_Na_ and I_Kr_ as well as suppress delayed after-depolarizations (DAD) in atrial tissue preparations by reducing calcium overload [41]. Use-dependent late sodium channel blockade has been demonstrated in atrial tissue, but minimally in ventricular tissue and purkinje fibers [42]. The atrial selectivity of Ranolazine provides the promise for a wide safety profile, but clinical experience is limited. There is also interest in combining Ranolazine with Dronedarone and Amiodarone to enhance the sodium-blocking effect on AF [43, 44].

Clinically, Ranolazine has been used for conversion of recent-onset AF, as well as cardioversion-resistant AF and for the prevention of post-operative AF [45-47]. The largest experience comes from the Metabolic Efficiency with Ranolazine for Less Ischemia in Non-ST elevation acute coronary syndromes (MERLIN) study, an anti-anginal study with Ranolazine in 6560 patients hospitalized for non-STEMI and acute coronary syndrome [48]. In this high-risk population, pre-specified analyses showed a reduction in non-sustained VT, SVT and a trend to reduced new onset AF (1.7% vs 2.4%, p=0.08). Further studies are needed before we will understand the role of this unique agent in the clinical management of AF, but the promise of a safe late sodium channel-blocking agent is welcome.

## FUTURE AGENTS:

With greater understanding of the molecular mechanisms underlying AF, there is increasing potential to alter the function of trans-membrane channels and intracellular channels in AF. A recent review has highlighted a number of novel agents that are under investigation [49]. The low potential for pro-arrhythmia makes each of these agents very interesting, but all are far from clinical use. Broadly speaking, agents are being developed to alter ryanodine receptor function at the sarcoplasmic reticulum to reduce calcium overload and associated DAD’s. Atrial selective potassium channel blocking agents (I_Kur_ blockers) and inward rectifier (I_KAch_) blocking agents as well as gap junction modifying agents are in varying phases of clinical trials.

With the list of promising agents noted above, there is reason to be hopeful for the improved pharmacological management of AF. Nonetheless, it should be noted that the last promising agent, Dronedarone, has been a decade in development at considerable cost. While it promised to be Amiodarone-like in its efficacy, but with fewer side effects, it has now been relegated to first-line treatment of a minority of patients with AF. Likewise, Vernakalant has not been FDA approved. Promising agents like Azimilde and Tedisimil have come and gone. At the same time, catheter ablation continues to make great strides. It remains to be seen if one or more of the novel atrial selective agents will be able to achieve the required high efficacy and safety threshold.

## Figures and Tables

**Table 1.** Antiarrhythmic Drugs in Atrial fibrillation

Drug	Mechanism	Efficacy^i^	Side effects	Contraindications^ii^	Comment
Amiodarone	Predominantly K^+^channel blocker but also Vaughan-Williams Class Ia, II and IV effects.	60-70%	Gastrointestinal (GI) upset, tremor, neuropathy, thyroid dysfunction, photosensitivity, pulmonary fibrosis, and hepatic toxicity	LQTS, severe pulmonary or liver disease.	Low risk of pro-arrhythmia. Toxicity is seen with higher doses over time. Metabolic interaction with digoxin and warfarin.
Sotalol	K^+^ channel (I_Kr_) and β blocker.	30-50%	Fatigue, bradycardia, dyspnea, GI upset, asthenia and TdP.	LQTS, LVEF <40%, CRF. Caution with diuretic use, and elderly.	Measure QT interval 1 week after initiation or dose adjustment.
Propafenone	Na^+^ channel blocker, (Class Ic) and weak β blocker effect.	30-50%	Potential to organize AF into Aflutter with 1:1 AV conduction, pro-arrhythmia, metallic taste, GI upset, bronchospasm, dizziness and agranulocytosis (rare).	IHD, impaired LV systolic function, AV conduction disease.	Use with AVN blocker.
Flecainide	Na^+^ channel blocker (Class Ic).	30-50%	Potential to organize AF into Aflutter with 1:1 AV conduction, pro-arrhythmia and dizziness.	IHD, impaired LV systolic function, AV conduction disease.	Use with AVN blocker.
Dofitilide	K^+ ^channel (I_Kr_) blocker (pure class III)	50-60%	TdP	LQTS, CRF	Initiate while monitored in hospital for at least 3 days.
Dronedarone	K^+^ (I_Kur_, I_Kr_, I_Ks_, I_K1_, I_To_), Na^+^, L-type Ca^2+^ channel and β blocker.	40%	GI upset and bradycardia. Elevated creatinine levels without affecting GFR.	LVEF <40%, CHF or permanent AF.	Raises digoxin levels. Reduces CV hospitalization and mortality in non-permanent AF.
Vernakalant	Atrial selective Na^+^ and K^+^(I_Kur, _I_KAch_) channel blocker	49% at 90 days	Bradycardia. Dysgeusia, sneezing, parasthesia and hypotension with intravenous administration.	Nil	Limited clinical data available outside short-term trials.
Ranolazine	Atrial selective I_Na_, I_Ca_, I_Kr_ and I_Ks_ channel blocker.	N/A	QT interval prolongation.	LQTS.	Indicated for treatment of angina.

Abbreviations: LQTS = Long QT syndrome, LVEF = Left ventricular ejection fraction, TdP = Torsade de pointes, CRF = Chronic renal failure, IHD = Ischemic heart disease, GFR = Glomerular filtration rate, CV = Cardiovascular, CHF = Congestive heart failure.

i Unless otherwise specified, efficacy refers to maintenance of sinus rhythm at 1 year.

ii Includes both absolute and relative contraindications.
